# Understanding Scapulohumeral Periarthritis: A Comprehensive Systematic Review

**DOI:** 10.3390/life15020186

**Published:** 2025-01-26

**Authors:** Daniel-Andrei Iordan, Stoica Leonard, Daniela Viorelia Matei, Dragos-Petrica Sardaru, Ilie Onu, Ana Onu

**Affiliations:** 1 Department of Individual Sports and Kinetotherapy, Faculty of Physical Education and Sport, “Dunarea de Jos” University of Galati, 800008 Galati, Romania; daniel.iordan@ugal.ro (D.-A.I.); leonard.stoica@ugal.ro (S.L.); 2 Center of Physical Therapy and Rehabilitation, “Dunărea de Jos” University of Galati, 800008 Galati, Romania; 3 Department of Biomedical Sciences, Faculty of Medical Bioengineering, University of Medicine and Pharmacy “Grigore T. Popa”, 700454 Iasi, Romania; daniela.matei@umfiasi.ro; 4 Department of Physiotherapy, Elipetro Med Clinic, 610119 Piatra Neamt, Romania; chirila.ana@d.umfiasi.ro; 5 Doctoral School, University of Medicine and Pharmacy “Grigore T. Popa”, 700454 Iasi, Romania

**Keywords:** rotator cuff lesions, impingement syndrome, calcifying tendinitis, bicipital tendonitis, adhesive capsulitis, functional assessment

## Abstract

Background: This systematic review examines the clinical presentations and prevalence of scapulohumeral periarthritis (SP) by synthesizing the relevant literature from open-access articles from international databases (Medline, Pedro, and EBSCO). Methods: Keywords guiding the review included ‘scapulohumeral periarthritis’, ‘clinical forms’, ‘incidence’, ‘impingement syndrome, ‘calcifying tendinitis’, ‘bicipital tendonitis’, ‘shoulder bursitis’, ‘adhesive capsulitis or frozen shoulder’, ‘rotator cuff tears’, ‘functional assessment’, and ‘clinical trials’. Eligible studies included randomized controlled trials, nonrandomized controlled trials, cross-sectional studies, and review articles published between 1972 and 2024. Results: Our screening identified 2481 initial articles, of which 621 were further reviewed for eligibility resulting in 107 articles that met the relevance criteria. The findings highlight six distinct clinical forms of SP, such as partial rotator cuff tears and calcific tendinitis, each characterized by specific pathological features and prevalence patterns. Key factors contributing to SP include injuries, scapular instability, acromion deformities, and degenerative rotator cuff changes. Functional assessments, including the Neer, Hawkins, Pain Arc, and Yocum tests, demonstrated diagnostic value in distinguishing SP from other shoulder conditions. Conclusions: By comprehensively analyzing the clinical forms, functional assessment methods, and prevalent lesions of SP, functional testing can improve early diagnosis and guide personalized physiotherapy protocols for optimal rehabilitation in the physiotherapist’s practice.

## 1. Introduction

The term ‘Scapulohumeral periarthritis’ (SP) has become somewhat outdated in contemporary medicine, as it fails to specify the exact pathology affecting the shoulder. Thanks to advances in diagnostic techniques, particularly highly sensitive and highly specific clinical tests and imaging, clinicians are now able to identify the exact pathologic condition causing shoulder pain and dysfunction. Instead of relying on the broad and imprecise term ‘periarthritis’, which in the past referred to inflammation of the soft tissues around the shoulder joint, modern diagnoses are based on a combination of clinical examination and imaging findings [1,2].

The so-called SP includes both lesions of the muscles and of the joint capsule itself [1]. In general, pain, limited movements, and muscle weakness from onset to several years are the symptoms that people diagnosed with this pathology suffer from, being an umbrella term covering several shoulder conditions [2]. It is usually present during mobilization/stretching in a large proportion of the population. A few years after the onset of the pathology, it presents with joint pain and stiffness according to Yang et al. (2008) [3].

A recent study analyzed costs associated with shoulder pain in 277 patients on an orthopedic waiting list in an Australian public hospital. It was found that the average annual healthcare cost per patient was AUD 7563, with additional productivity losses increasing costs to AUD 22,378 per year for those employed. Over two years, inpatient care costs averaged AUD 3835.78 per patient, with outpatient services accounting for 51% of these costs [4]. Alqahtani et al. (2022), in a cross-sectional study, investigated the prevalence of shoulder pain and awareness of Adhesive Capsulitis (AC) among 505 respondents. The results showed that 59.4% of the respondents were male, 44.6% were employed, and 69.31% were aware of AC, with 38.6% believing that those over 60 years of age were most affected [5]. Alghamdi et al. (2024), in a cross-sectional study, investigated the prevalence of shoulder pain and AC awareness among 378 participants in Saudi Arabia. The prevalence of shoulder pain was 32.8%, with significant associations found with aging (35–44 years), diabetes, retirement, and other comorbidities [6].

AC affects 2–5% of the Swedish population, especially people aged 40–60, and typically goes through three phases: painful, stiff, and recovery, with an average duration of 30 months. Treatment often includes pain therapy and physiotherapy, with more severe cases benefiting from interventions such as manipulation under anesthesia, arthrography by distension arthrography, or arthroscopic release to speed functional recovery [7]. A recent study analyzed risk factors for AC in 1205 patients in the Xinjiang region of China between 2018 and 2023. The analysis showed that female sex and a history of diabetes were independent risk factors for AC, while poor sleep quality and constipation were negatively associated with its occurrence [8]. Lyne et al. (2022), in a qualitative study explored the lived experience of people with AC, focusing on its physical, psychological, and socioeconomic impact. Through ten semi-structured interviews, five key themes emerged: severity of pain, loss of independence, an altered sense of self, significant psychological effects, and varied experiences with healthcare providers. The study emphasizes that AC has profound effects beyond being a self-limiting condition, highlighting the need for comprehensive management that addresses both physical and emotional aspects [9].

SP complaints are common in the UK, with an estimated prevalence of 14%, and 1% to 2% of adults consulting their general practitioner annually for new-onset shoulder pain. The socioeconomic burden of SP is significant, impairing work and daily activities, leading to lost working hours. Shoulder problems account for 2.4% of all GP consultations in the UK and 4.5 million physician visits annually in the USA, with shoulder pain management in the USA costing an estimated USD 3 billion annually. AC, a condition with a cumulative incidence of 2.4 per 1000 people annually, was first described in 1875 and is characterized by inflammation and scarring of the shoulder capsule, leading to restricted movement [10]. Rangan et al. (2020) conducted a multicenter randomized trial in which 503 participants with primary AC were assigned to manipulation under anesthesia, arthroscopic capsular release, or early structured physiotherapy. Treatments were assessed using the Oxford Shoulder Score (OSS) at 12 months. The OSSs were 38.3 for manipulation, 40.3 for capsular release, and 37.2 for physiotherapy, showing only small differences between groups. The mean differences between the groups were small, with manipulation under anesthesia being the most cost-effective intervention based on a willingness-to-pay threshold of GBP 20,000 per quality-adjusted life-year [11]. Laslett et al. (2007) conducted a study to investigate shoulder pain, disability, and quality of life (QoL) in diabetic patients compared to a non-diabetic control group over 12 months. The results revealed that 35% of diabetics and 17% of controls had current shoulder symptoms. Shoulder pain and disability were more pronounced in diabetic patients, and both worsened over time, with one in three diabetics experiencing shoulder symptoms compared to one in six non-diabetics [12].

A retrospective cohort study in Switzerland analyzed the socioeconomic impact of AC, particularly in terms of work absence and treatment costs following shoulder injuries. The study found that 5% of 456,926 patients with shoulder injuries developed post-traumatic AC. These patients had significantly longer periods of sick leave, with 30.8% experiencing long-term work disability and 9.7% experiencing very long-term disability, compared with 9.4% and 1.3% in cases without AC. Treatment costs for post-traumatic AC were significantly higher, averaging CHF 34,000 per case compared with CHF 8000 for non-AC injuries. The total annual cost for AC cases covered by compulsory accident insurance was CHF 78 million, excluding idiopathic cases [13].

The shoulder is frequently considered a difficult joint to assess, mainly due to its complexity and the large number of clinical tests available to detect one of the forms of this condition. At least 109 distinct tests for shoulder assessment are included in the literature, providing valuable information about the diagnostic accuracy and clinical applicability of the different tests used in shoulder injuries according to Moen et al. (2010) [14]. Other authors state that there are 184 shoulder assessment tests, of which only 10 are the most effective and commonly used by specialists [15].

According to Naredo et al. (2002), the Neer, Hawkins, and Yocum tests are used to diagnose subacromial impingement syndrome, and the other seven tests such as Jobe’s Test (supraspinatus), Gerber’s Test (subscapularis elevation and strength assessment), Patte’s Test (infraspinatus and teres minor), Yergason’s Test, Supination Palm Test, and Popeye’s Sign (biceps) are performed to prove the presence of tendon disorders [16].

Thus, we reviewed the most effective and applied tests to provide clinicians with sufficient information on the effective and early detection of certain shoulder injuries and photographs of the practical testing procedure, aiming at correct performance. Also, imaging investigations for rotator cuff injuries, instabilities, labral tears, and acromioclavicular disorders can provide false-positive or false-negative results. For this reason, it has been suggested that traditional clinical examination/functional assessment of the shoulder should continue to be the central element of diagnosis [17,18].

This review aims to explore various shoulder pathologies, including their clinical features and diagnostic physical tests. It will also delve into how the definition of SP has evolved in clinical practice, providing a deeper understanding of the shoulder’s complex anatomy and the conditions that affect it. Through this examination, the review will clarify how precise diagnoses can guide appropriate treatment options, moving away from the vague concept of “periarthritis” and offering a more targeted approach to shoulder care.

**H1.**  *It is assumed that by analyzing and interpreting literature information on the clinical forms of SP we will be able to identify the most prone lesion.*

**H2.**  *The importance of functional assessment in the detection of clinical forms of SP is to help us in the efficient and correct detection of each disorder for a future physiotherapy protocol.*

## 2. Methods

### 2.1. Systematic Search Strategy

For this systematic review, we searched for open-access articles in the fields of physical therapy and exercise physiology relevant to SP. Searches were performed across international databases: Medline, Pedro, and EBSCO. Given the specific focus of this review, a total of 107 articles, published between 1972 and 2024, were selected. To enhance our systematic review’s transparency and methodological rigor, we adhered to the guidelines outlined in the PRISMA (Preferred Reporting Items for Systematic Reviews and Meta-Analyses) declaration. Our systematic review follows the PRISMA checklist, detailing each literature selection and screening phase, from initial identification to final inclusion. This includes the number of records identified through database searching, duplicates removed, records screened, full-text articles assessed for eligibility, and studies ultimately included in the review. Following PRISMA guidelines, we present a flow diagram to visually depict each selection process step, providing a clear and transparent overview of our methodology.

### 2.2. Inclusion and Exclusion Criteria

The articles selected for this review adhered strictly to the specified methodology. Eligibility criteria required that all relevant keyword combinations appear within the article’s keywords. The following types of studies were included: cross-sectional studies, non-randomized controlled trials, randomized controlled trials, observational studies, and review articles. Articles written in English have been accepted in other languages (e.g., Russian, Swedish, and German). This study focuses on scapulohumeral periarthritis, including impingement syndrome, calcifying tendinitis, bicipital tendonitis, shoulder bursitis, adhesive capsulitis/frozen shoulder, and rotator cuff tears with an emphasis on functional assessment and clinical tests for accurate diagnosis and evaluation.

Exclusion criteria were as follows: studies with fewer than fifteen participants, duplicate publications or datasets, studies lacking functional assessment or shoulder-specific diagnostic tests, and articles deemed irrelevant to the study’s focus based on the predefined inclusion criteria.

PICO criteria:
Population (P): Adults with scapulohumeral periarthritis conditions, including impingement syndrome, calcifying tendinitis, bicipital tendinitis, shoulder bursitis, adhesive capsulitis (frozen shoulder), and rotator cuff tears.
Intervention (I): Functional assessment methods and clinical diagnostic tests specific to shoulder conditions.
Comparator (C): Studies comparing different diagnostic or functional assessment methods, when applicable.
Outcome (O): Diagnostic accuracy, functional assessment reliability, and clinical relevance of shoulder-specific tests.

## 3. Results

A total of 2481 articles were identified through searches of Medline (589 articles), Pedro (138 articles), and EBSCO (1754 articles) databases, using the following search strategy: “impingement syndrome” (MeSH Terms) OR [“impingement” (All Fields) AND “syndrome” (All Fields)] “calcifying tendinitis” (MeSH Terms) OR [“calcifying” (All Fields) AND “tendinitis” (All Fields)] “bicipital tendonitis” (MeSH Terms) OR [“bicipital” (All Fields) AND “tendonitis” (All Fields)] “shoulder bursitis” (MeSH Terms) OR [“shoulder” (All Fields) AND “bursitis” (All Fields)] “adhesive capsulitis” (MeSH Terms) OR [“adhesive” (All Fields) AND “capsulitis” (All Fields)] AND “frozen shoulder” (MeSH Terms) OR [“frozen” (All Fields) AND “shoulder” (All Fields)] “rotator cuff tears” (MeSH Terms) OR [“rotator” (All Fields) AND “cuff ” (All Fields) AND “tears” (All Fields)] “scapulohumeral periarthritis” (MeSH Terms) OR [“scapulohumeral” (All Fields) AND “periarthritis” (All Fields)] “functional assessment” (MeSH Terms) OR [“functional” (All Fields) AND “assessment” (All Fields)] “clinical test” (MeSH Terms) OR [“clinical” (All Fields) AND “test” (All Fields)] (Supplementary Materials Files S1).

**Figure 1:**
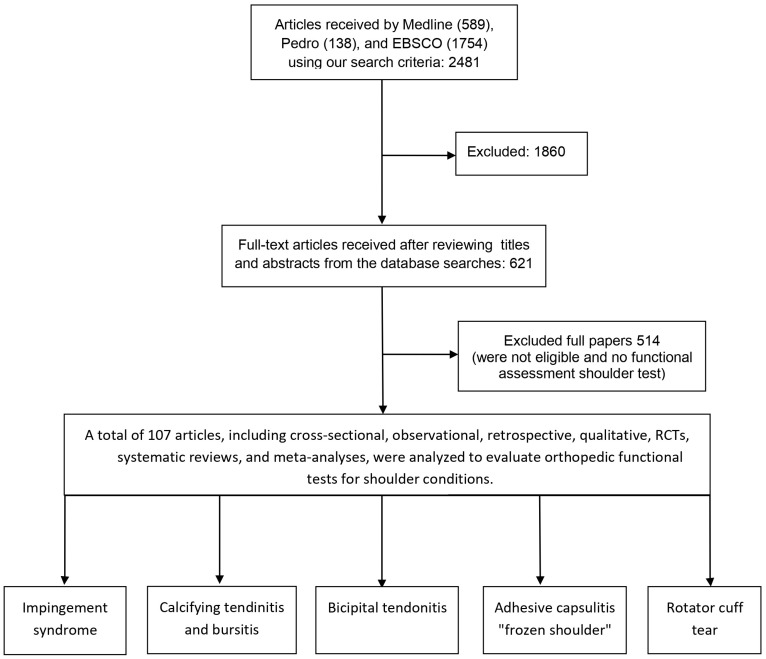
PRISMA flow diagram of the article selection process for studies.

After initial screening, 1860 articles were excluded based on title and abstract review. The remaining 621 full-text articles were evaluated for eligibility. Following further evaluation, 514 additional articles were excluded because of a focus on conditions outside the specified domains and keywords. One hundred and seven bibliographic entries met the criteria for bad relevance based on the specified keywords (Figure 1) (Supplementary Materials Files S2).

### Clinical Form, Prevalence SP

According to Mangold (2023), there are six clinical forms categorized under the term SP, each associated with a certain prevalence of lesions (Table 1) [19]:

Impingement Syndrome (IS) is one of the most common forms of SP, with prevalence rates ranging from 7% to 34%**.** The wide range reflects variability in diagnostic approaches and patient demographics. According to studies by Castaldo et al. (2023) and Grassi et al. (2012), IS is typically caused by mechanical compression of the rotator cuff structures beneath the coracoacromial arch, often leading to pain and functional limitations [20,21].

Calcifying Tendinitis (CT) affects between 2.7% and 20% of individuals, with 10% to 20% of cases presenting bilaterally. This condition, characterized by calcium deposits in the rotator cuff tendons, is often seen in middle-aged adults. Research by Dudani et al. (2023), Sansone et al. (2018), and Oliva et al. (2012) highlights its symptomatic variability, with some patients experiencing significant pain and others remaining asymptomatic [22,23,24].

The incidence of Degenerative Tendinitis increases proportionally with patient age and the progression of rotator cuff disease. Ahrens and Boileau (2007) emphasize the age-related decline in tendon integrity as a key factor contributing to the prevalence of this condition [25].

Bicipital Tendinitis (BT) is rare, occurring in approximately 5% of cases. As described by Ahrens and Boileau (2007), this condition involves inflammation of the long head of the biceps tendon, often coexisting with other SPs, such as rotator cuff pathology or shoulder instability [25].

AC has a prevalence of 7.1%**,** according to Stella et al. (2022). It is characterized by progressive stiffness and pain, often leading to significant functional impairment. Early intervention and physiotherapy are essential to restoring mobility [26].

Rotator cuff tears (RCT) are notably common, affecting 25% of individuals over 50 years old and 20% of those over 20 years old. Studies by Minagawa et al. (2006) and Yamamoto et al. (2010) reveal an age-dependent increase in prevalence, correlating with degenerative changes in tendon structure. These tears are a leading cause of chronic shoulder pain and dysfunction, often necessitating surgical intervention [27,28].

## 4. Epidemiology of Clinical Forms of SP

### 4.1. Impingement Syndrome

According to Habermeyer et al. (2010), subjects presenting with this condition are over 40 years of age and endure persistent pain, but without having had any previous known injury [29]. Pain is felt when lifting the affected limb between 70° and 120°, also nicknamed the “painful arch”, when forcibly bringing the arm above the head, and also when lying supine on the diseased side [30,31]. Some external factors that may influence the occurrence of IS are exposure to heavy weights, smoking, and infections [32].

A localized pain in the anterolateral acromial area radiating to the middle of the lateral humerus is a typical sign of subacromial IS [33]. Some patients also present with a proprioceptive defect affecting their musculature and kinematic movements of the shoulders [34,35].

IS is defined as the compression of the rotator cuff as well as the subacromial bursa. It is one of the common causes of shoulder pain, in orthopedic practice and is present in 65% of cases [36,37]. It is estimated that 50% of people diagnosed with this pathology appear to fully recover within the first 12 months [38].

The basis of the diagnostic evaluation is the history and physical examination and 90% of the sensitivity is diagnosed following physical examination [39].

According to Escamilla et al., (2014), three stages of IS have been described [40]:

(i). The first stage presents with edema, hemorrhage, painful discomfort caused by inflammation of the supraspinatus tendon and the long head of the biceps brachii and is seen in people younger than 25.

(ii). Stage two involves fibrotic changes in the supraspinatus tendon and the subacromial bursa, targeting subjects aged 25–40 years, who experience pain with active movements.

(iii). The third stage is characterized by shoulder pain, partial or total rotator cuff rupture, and osteophyte formation and affects people over 40.

According to Koester et al. (2005), subacromial IS encompasses a range of subacromial space pathologies [33] such as the following:

- Calcified tendinitis;
- Subacromial bursitis;
- Rotator cuff tendinitis;
- Partial RCT, all of which can only be distinguished by MRI, or arthroscopy.

Several conditions have been associated with SIS, namely, the abnormal position of the scapula and asymmetry of more than 1 cm in scapular protraction (combination of external rotation and superior translation) between both shoulders. Therefore, MRI has confirmed the influence of protraction on the shrinkage of the subacromial space [40].

According to Dickens, Williams, and Bhamra (2005), the occurrence of IS is caused by several factors, including poor posture, acromion deformity, overuse, scapular instability, glenohumeral joint hypermobility, and rotator cuff degeneration [41]. For people suffering from IS, eccentric-type exercise is beneficial and effective as it places greater demands on the muscular system, stimulating the elongation of muscle fibers through a control on the return (“negative”) moment of the exercise in the subacute phase implemented by the physiotherapist [42,43,44].

### 4.2. Calcifying Tendinitis and Bursitis

Calcifying tendinitis (CT) is another acute or chronic, painful shoulder condition that pathogenetically is unclear, but is thought to be due to calcium deposits around or within the rotator cuff tendons [45] following the metaplastic transformation of tenocytes to chondrocytes [46].

CT results from reduced oxygen delivery to poorly vascularized tendons, leading to metabolic changes that cause tenocytes to transform into chondrocytes. These chondrocytes produce a cartilaginous matrix that eventually calcifies. The condition progresses through precalcific, calcific, and postcalcific stages, with the calcific stage further divided into formative, resting, and resorptive phases. During the resting phase, hard calcifications form and displace tendon fibers, while the resorptive phase involves soft calcifications rupturing under mechanical forces like tension and compression. Hydroxyapatite crystals can migrate within tendons through delaminating zones, moving to various anatomical locations, including the bursa, muscle, bone, and joint spaces. These migratory patterns are driven by mechanical forces and can be visualized using ultrasound, which shows hyperechoic foci in short-axis views. The calcifications can migrate to sites such as the subacromial bursa, supraspinatus muscle, and the glenohumeral joint. Compression and tension forces facilitate the rupture and dispersion of calcifications through tendon layers [47].

CT predominantly affects people aged 40 to 60, with a higher prevalence in women. Interestingly, it does not necessarily correlate with heavy manual labor. Approximately 90% of calcifications occur in the infraspinatus and supraspinatus tendons [22].

According to Gosens et al. (2009), three stages of CT have been described [48]:

1. The precalcifying stage: the subject is asymptomatic and is described by fibrocartilaginous metaplasia of the tendon.

2. The calcifying stage has two phases: formation and resorption. In the formative phase, calcium hydroxyapatite crystals are deposited in the tendon, and in the resorptive phase there is edema, an increase in intra tendinous pressure, and osteophyte penetration into the subdeltoid subacromial bursa, pain may not respond to analgesics.

3. The postcalcification stage begins with the resorptive phase, granulation tissue formation, and tendon remodeling by fibroblasts, with complete recovery that may last several months.

Subjects suffering from scapulothoracic bursitis present with pain and cramping at the scapular level especially when positioning the arm above the head in daily activities [49]. Authors Hirji, Hunjun, and Choudur (2011), state that bursitis is the inflammation of the bursitis, being positioned between the inferior surface of the acromion to the deltoid muscle and the acromioclavicular joint and between the rotator cuff tendons [50].

According to Faruqi and Rizvi (2019), bursitis presents with symptoms such as decreased joint mobility, pain in the upper shoulder region, and local edema associated with redness to diagnose it objectively it is necessary to use MRI or Musculoskeletal ultrasonography [51]. Secondary bursal inflammation following trauma or overuse due to work or sports activities is called scapulothoracic bursitis [52].

Two major or anatomic and four minor or adventitial bursae are described in the scapulothoracic joint (Warth, Spiegl, and Millett, 2015) [53]. The infraserratus bursa (the first major bursa) is located between the thoracic wall and the serratus anterior muscle [54]. The supraserratus bursa (second bursa) is located between the serratus anterior muscle and the subscapularis muscles; minor bursae occur in response to abnormal biomechanics of the scapulothoracic joint [55].

### 4.3. Bicipital Tendonitis

Degenerative tendinopathy of the long head of the bicep’s tendon presents with hemorrhagic adhesions, scarring, tendon thickening, disorganization, and tissue irregularity [56] and is caused by repetitive rubbing and sliding of the long head of the bicep’s tendon in the intertubercular groove [57].

According to Ahrens and Boileau (2007), the incidence of BT is directly proportional to the extent of rotator cuff disease but also the age of the patient, and the presence of this condition is best evidenced by MRI as well as Doppler ultrasound [25].

BT is the inflammation of the long head of the biceps and is a pain-producing condition of the shoulder [58]. Primary BT occurs in 5% of cases and is constantly accompanied by rotator cuff injuries or IS [59].

Genç and Duymaz (2020), included 80 patients in their study, diagnosed with BT using Speed test, Yergeson test, biceps groove tenderness, and MRI evaluation [60]. Out of the study, 62.5% percent were females and 37.5% were males, BT was present in 61.9% of the right arm. The non-conjugation method is the first treatment to be followed for the recovery of BT, followed by conservative treatment including activity modification, non-steroidal anti-inflammatory drug therapy [61], rest, and physiotherapy [62].

### 4.4. Adhesive Capsulitis—“Frozen Shoulder”

Another shoulder condition is AC, which produces a limitation of movement due to progressive fibrosis as well as contraction of the glenohumeral joint capsule, the etiology remains unclear [63,64], but is clinically and paraclinical diagnosed by history, physical examination, as well as imaging [65].

In 1934, Codman described “frozen shoulder” as a syndrome that is “difficult to define, difficult to treat, and difficult to explain”, a fact also confirmed by Ryan et al. (2016) [66]. An AC is considered a benign pathology with a recovery that can span up to two years. This syndrome rarely occurs before the age of 40, being commonly seen between the ages of 40 and 60 and considered uncommon in people over the age of 70 [2,67]. In addition, women are more prone than men to this condition [68].

However, the diagnosis of AC can be made through typical imaging appropriateness; they demonstrate the benefits of ultrasound in identifying shoulder problems which are popular in the literature [69,70].

It is estimated that between 7% and 15% of patients permanently lose some mobility, but only a few suffer persistent functional disability. According to the literature, 50% of subjects continue to experience mild discomfort even after seven years, while 60% remain with stiffness, thus phytotherapy can be of great help in relieving persistent symptoms, as 40% of patients experience these problems [2,71].

Stella et al. (2022) included 1486 patients suffering from shoulder pain and stiffness in their study; 106 patients underwent an orthopedic evaluation to rule out other disorders and 30 subjects did not present with a definite diagnosis recommended further investigations such as MRI or radiography to rule out other alternative disorders [26]. They also showed that the subjects included in the study, and more specifically, one-third of them had several factors leading to the development of AC, such as Dupuytren’s disease, diabetes mellitus, thyroid disease, calcific tendinitis of the rotator cuff, and upper limb immobilization [26]. Of the 1486 subjects, 7.2% were clinically proven to have AC, 35 males and 71 females aged 55 years.

The clinical diagnosis is also based on the loss of range of motion when performing external rotation, and abduction that cannot reach 90°. It is noted that there is also a limitation of internal rotation and flexion [72]. External rotation is the best movement following which clinical severity is seen [73,74].

According to Nicholson (2003), another key symptom in the diagnosis of an AC is pain that intensifies during the night [72]. These aspects were also confirmed by authors Robinson et al. (2012) [2] and Kelley et al. (2013) [75], who identified this characteristic pattern specific to mobility limitation. They also emphasized the importance of excluding other conditions through appropriate imaging.

Diabetes, shoulder injuries, thyroid diseases, regional corneal pain syndrome, Dupuytren’s syndrome, Parkinson’s disease, cancer, and nephrolithiasis are risk factors in the development of AC [76]. These pathologies, along with a sedentary lifestyle and the partial or total absence of specific physical exercises to increase ROM, especially in the non-dominant shoulder, shows that AC syndrome is systemic [77].

The literature highlights that diabetes mellitus is the condition most commonly associated with AC, with the association between them estimated to be as high as 75.1% [67,78].

### 4.5. Rotator Cuff Tear

The general population has been surveyed indicating that RCT has a prevalence of 25% in people over 50 years of age [27], and 20% in subjects over 20 years of age [28], and one of the major causes is reported to be inflammation [79] and supraspinatus muscle injury [80].

According to Kobayashi et al. (2004), 20% of the population suffering from shoulder pain present to specific clinics [81], while the rest go to chiropractors, purchase painkillers, or ignore it, which means that 1 in 15 people with RCT come to a specialized clinic [82].

According to Itoi et al. (2006), 157 people presented to their clinic of which 138 patients (87.9%) came because of pain: 17 (10.8%) had pain and muscle weakness grade 3, and 2 subjects (1.3%) arrived because of decreased function and strength that was less than the value of 3 [83].

From an etiopathogenic point of view, there are several theories regarding etiology, but they have been categorized into “extrinsic” and “intrinsic” factors [84], and according to an older study by Neer (1972) [85], the most well-known extrinsic factor of RCT is IS, and in asymptomatic patients, tendon changes are common in diabetic personae [86,87].

Diabetic patients have restricted movement-specific joint ROM, and have an increased incidence of recurrence after surgery, numerous complications, as well and infections [88,89].

According to Via et al. (2013), there are three distinct acromial forms [84]:

- Type I or flat acromion;
- Type II or curved;
- Type III or hooked acromion, is associated with RCT in 70% of cases.

According to Galatz et al. (2006), nicotine harms the healing process of the injured tendon, and smokers are further away from positive results in cuff repair [90].

## 5. Functional Assessment Tests Shoulder

Functional assessment is a referral process in physiotherapist practice based on inspection; the affected arm is positioned laterally in the abduction and internal rotation and there is mild atrophy of the deltoid and supraspinatus muscles. On palpation, the subject shows tenderness over the trapezius muscle, the interscapular area, and the glenohumeral joint [91].

The external rotation is important in confirming the diagnosis because if it is impossible to perform an active as well as a passive movement, then the test is positive.

In cases where the patient can perform external rotation with relative ease, particularly with the assistance of a physician or physiotherapist, the diagnosis often leans toward an RCT. This distinction is essential, as an RCT typically demands a different treatment strategy, potentially involving specific physiotherapy exercises, lifestyle adjustments, or, in more severe instances, surgery [92].

Impingement tests

### 5.1. Neer Test

**Figure 2:**
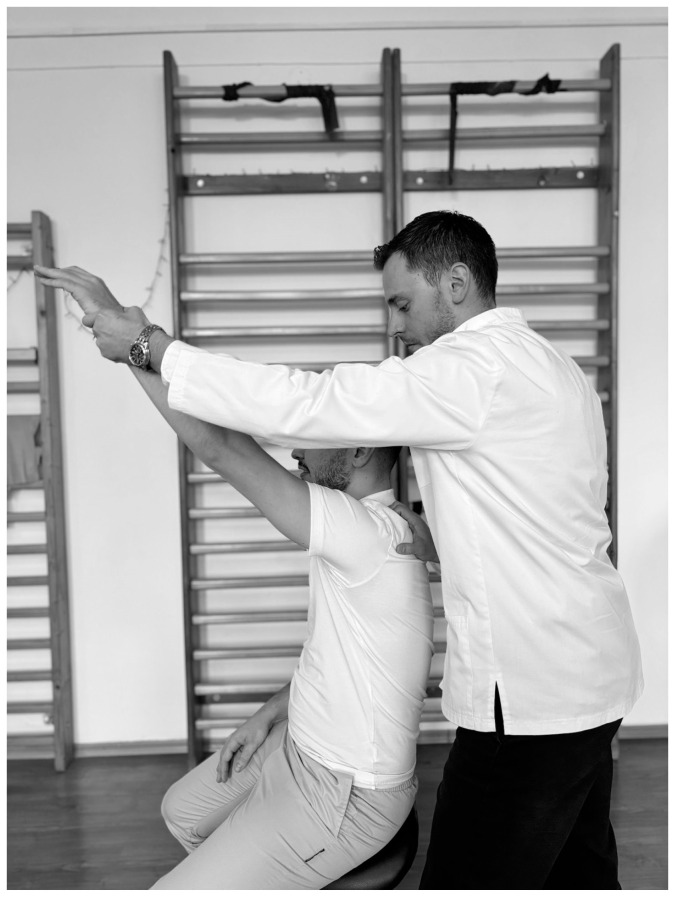
Neer test.

In physiotherapists’ practice, functional assessment is of reference; therefore the Neer test (Figure 2) is one of the most conclusive in IS, performed as follows: the physiotherapist stands behind the subject and holds the scapula with one hand to avoid scapular rotation, then raises the subject’s arm with the other hand to realize abduction and anteversion [93] and if the pain occurs before full flexion of the arm, the test is considered positive [94], with sensitivity ranging from 64% to 81% and specificity from 10% to 95% [95,96].

### 5.2. Hawkins Test

**Figure 3:**
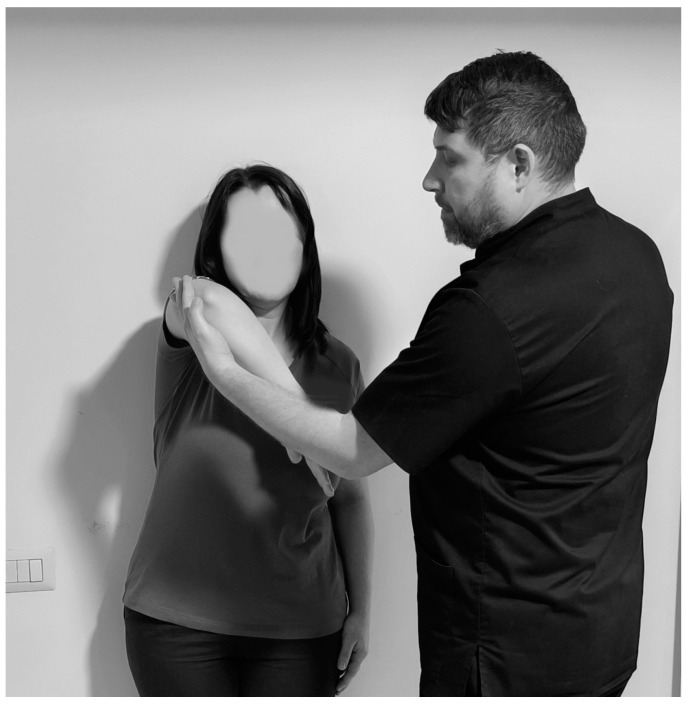
Hawkins test.

Another test that highlights the IS is the Hawkins test (Figure 3). To perform this test, the physiotherapist stands to the side of the subject and flexes the arm to 90°, with the elbow bent to 90° then the arm is bent slightly inward, and if pain occurs then the test is considered positive, the sensitivity ranging from 46% to 87% and specificity from 29% to 89% [95,96].

### 5.3. Pain Arc Test

**Figure 4:**
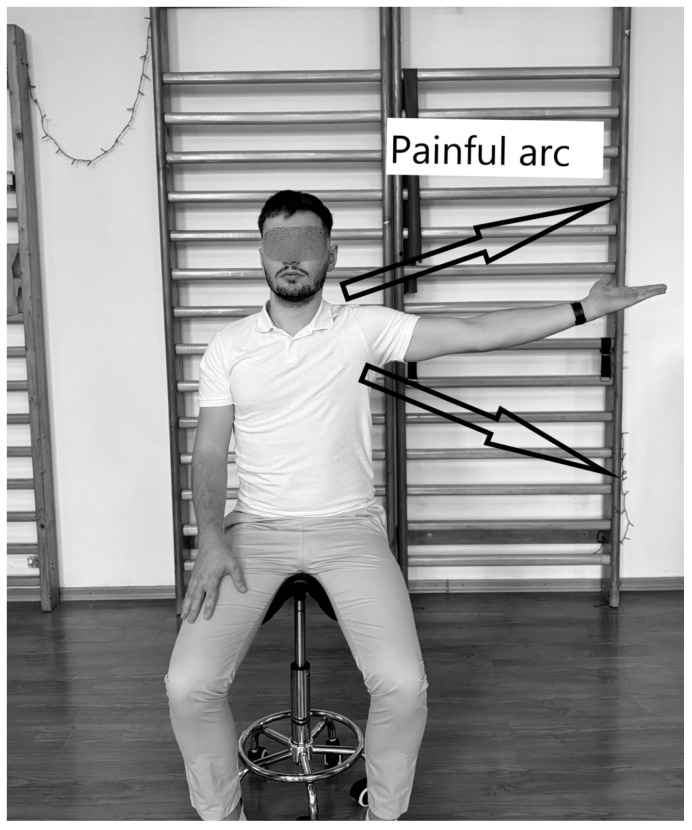
Pain Arc Test.

IS can also be diagnosed with this test (Figure 4), in which the physiotherapist instructs the subject, standing or sitting, to perform shoulder abduction between 60° and 120°. The test is considered positive if the subject feels pain within this range, indicating tendon and muscle inflammation [97].

### 5.4. Yocum Test

**Figure 5:**
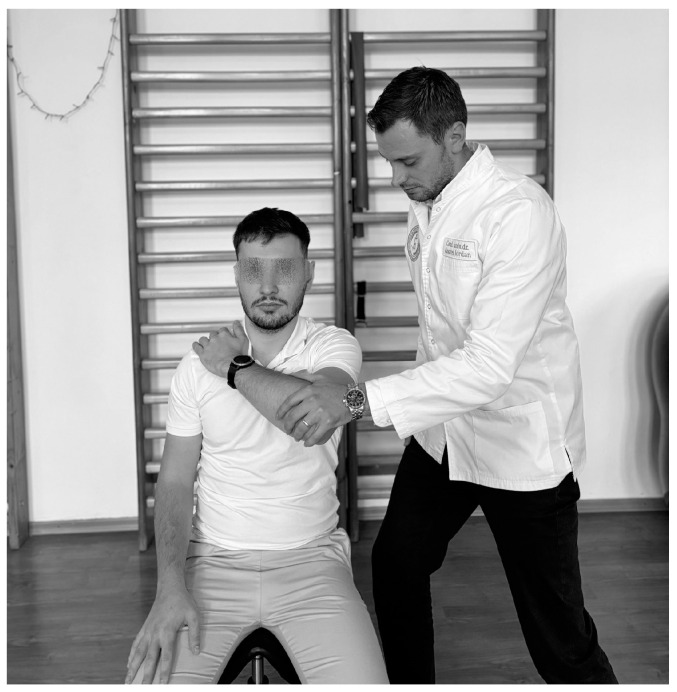
Yocum test.

To identify IS, the subject is required to be in a sitting position, the palmar face of the affected limb is positioned on the opposite shoulder, and then they attempt to raise the elbow of the affected shoulder without compensation (Figure 5). The physiotherapist stands to the side applying pressure. If pain is felt during the test, it is scored positive. The sensitivity of the test varies between 70% and 79% and the specificity between 40% and 92% [94,96].

Rotator cuff tests

### 5.5. Jobe Test

**Figure 6:**
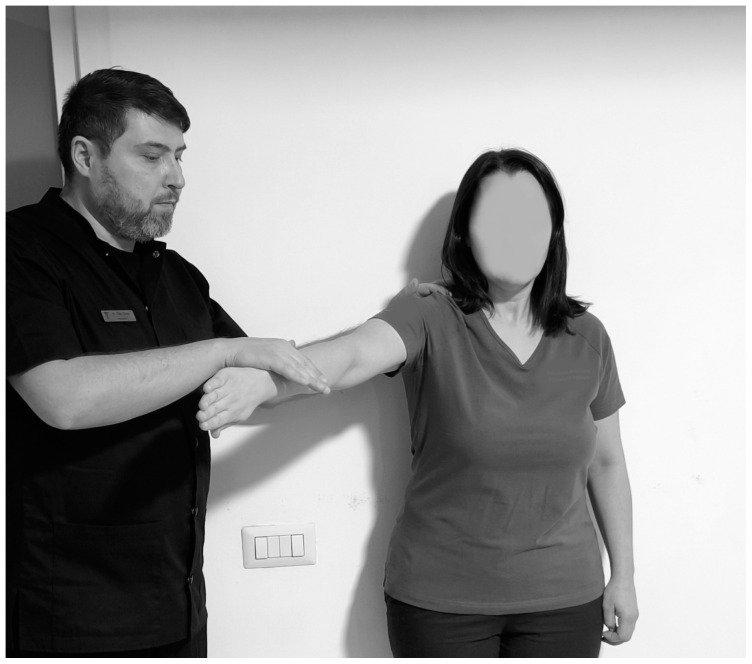
Jobe test.

The Jobe test (Figure 6) is conclusive for supraspinous and RCT. The subject sits while the physiotherapist stands to the side. The subject’s arms are abducted horizontally in the scapular plane 90°, with 30° of downward rotation and fingers pointing towards the floor. The physiotherapist then pushes the upper limb downwards while the subject tries to maintain the position [6]. The test is positive if pain occurs, with sensitivity ranging from 71% to 74% and specificity from 30% to 74% [96,98].

### 5.6. Patte Test

**Figure 7:**
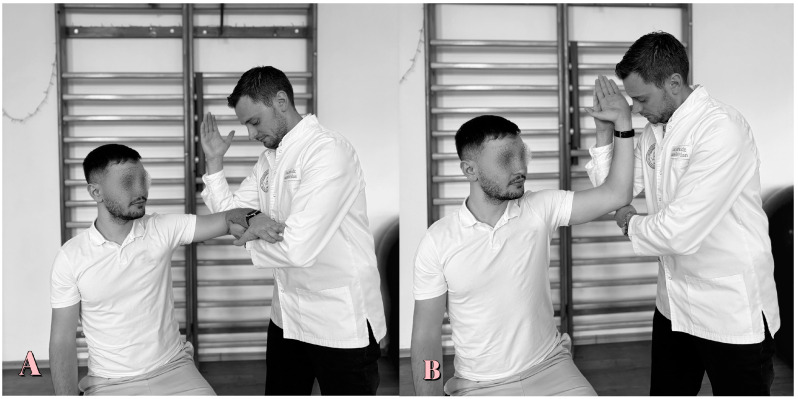
Patte test. (**A**) Initial position; (**B**) final position.

The Patte test (Figure 7) is conclusive for infraspinatus and teres minor and RCT performed with the subject in orthostasis, with the assessor facing the subject. The subject’s upper limb is flexed at 90° in the frontal plane, with elbow flexion of 90° but without external rotation. The subject is required to hold the arm up, and if this is not possible, the test is scored as positive. The sensitivity ranges from 70% to 99% and the specificity from 61% to 80% [99].

Regarding the Jobe and Patte tests, there are three guidelines: pain-free which indicates that the tendon shows no damage; endurance despite pain resulting in tendinitis; and inability to maintain gradual descent of the upper limb, which is as a sign of tendon rupture [96,99].

### 5.7. Gerber Test

**Figure 8:**
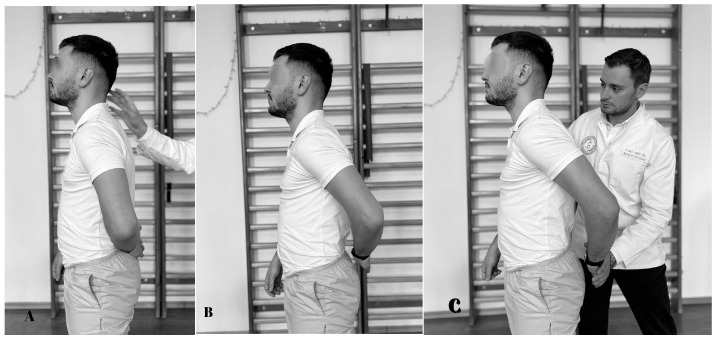
Gerber test. (**A**) Initial position; (**B**) the subject pushes back; (**C**) final position.

The test is conclusive for the assessment of subscapularis resistance and begins with the subject in orthostasis. The assessor stands behind the subject (Figure 8). The subject’s upper limb is positioned on the lower back, with the dorsal side of the palm touching the lumbar area, with the elbow bent at 90°. Then, the subject attempts to raise the hand from behind the back. The test is positive when the hand cannot be removed from the back which suggests that the subscapularis tendon is torn. If the subject succeeds in raising the hand, they are asked to maintain the position while the physiotherapist applies pressure to assess muscle strength [100]. The sensitivity is 6–68% and the specificity is between 23 and 90% [95,101].

Adhesive capsulitis

### 5.8. The Coracoid Pain Test

**Figure 9:**
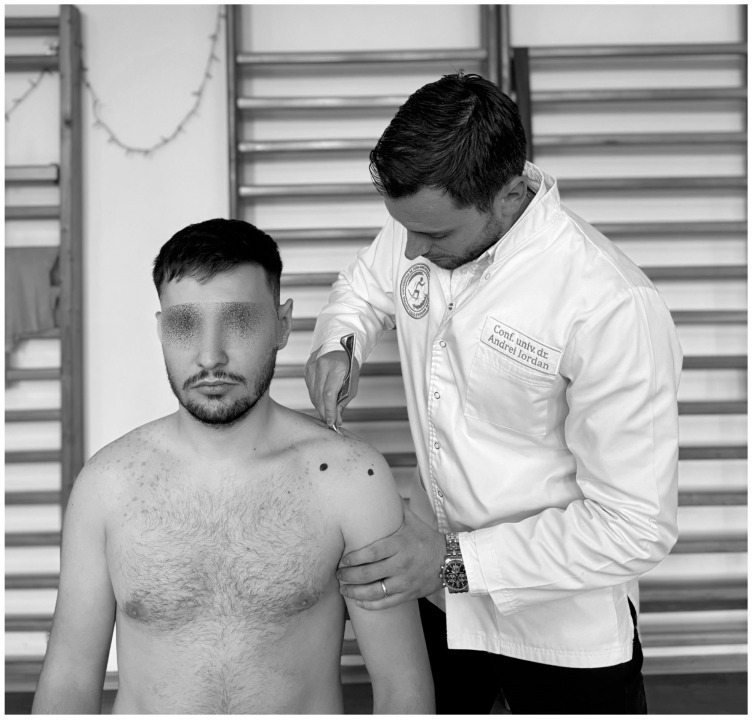
The coracoid pain test.

To identify the AC, a conclusive test would be the coracoid pain test (Figure 9). Thus, according to Carbone et al. (2010), palpation that causes pain perception may be a pathognomonic sign of an AC [102].

During the Coracoid Pain Test, pressure is applied to three reference areas: the coracoid process, the acromioclavicular joint, and the anterolateral subacromial area. The test is positive when the pain at the coracoid process scores at least 3 on the Visual Analog Scale (VAS), ranging from 0 (no pain) to 10 (most severe pain), compared to the other two areas. The test is performed first on the affected shoulder and then on the healthy shoulder [102,103].

### 5.9. Glenohumeral Internal Rotation Deficit

**Figure 10:**
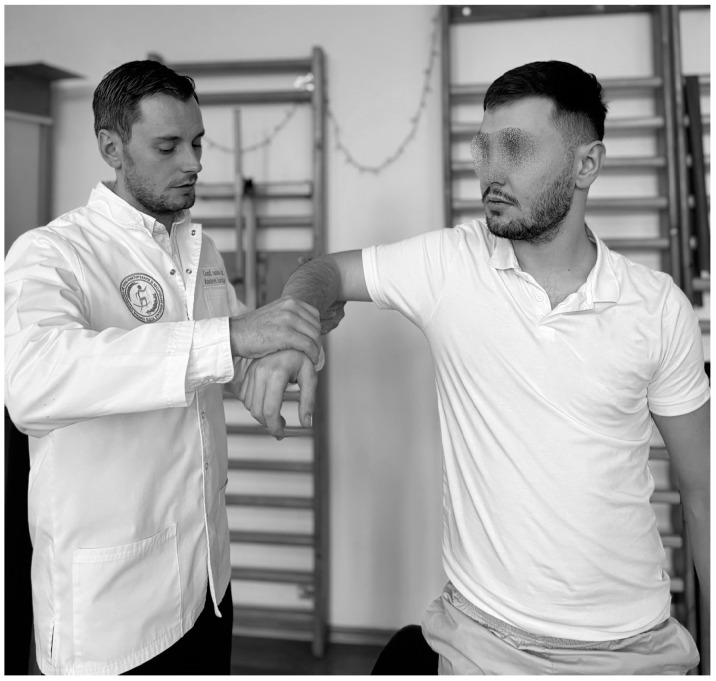
Glenohumeral internal rotation deficit.

Another important test (Figure 10) for the clinical detection of SP would be the limited passive internal rotation of the glenohumeral joint, so in the early stages, AC may present with the same symptoms as IS but later presents with progressive loss of motion, the amplitude of internal rotation (Koester et al., 2005) [33].

Bicipital tendonitis Tests

### 5.10. Yagerson Test

**Figure 11:**
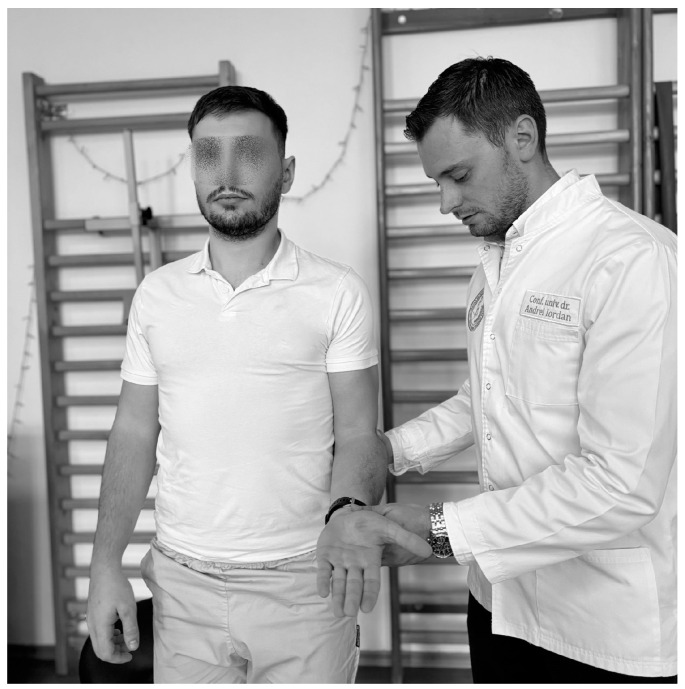
Yagerson test.

The Yagerson test (Figure 11) is used to confirm bicipital tendonitis. Thus, it is performed with the patient in orthostasis, the physiotherapist next to the subject, with the arm at the side and the elbow flexed at 90°, and the person is asked to keep the limb in supination against the pressure applied by the physiotherapist to bring it into pronation and the test is positive if they feel pain in the bicipital groove region [101]. The sensitivity ranges from 14% to 75% and the specificity in the test from 70% to 89% [95].

### 5.11. Speed Test

**Figure 12:**
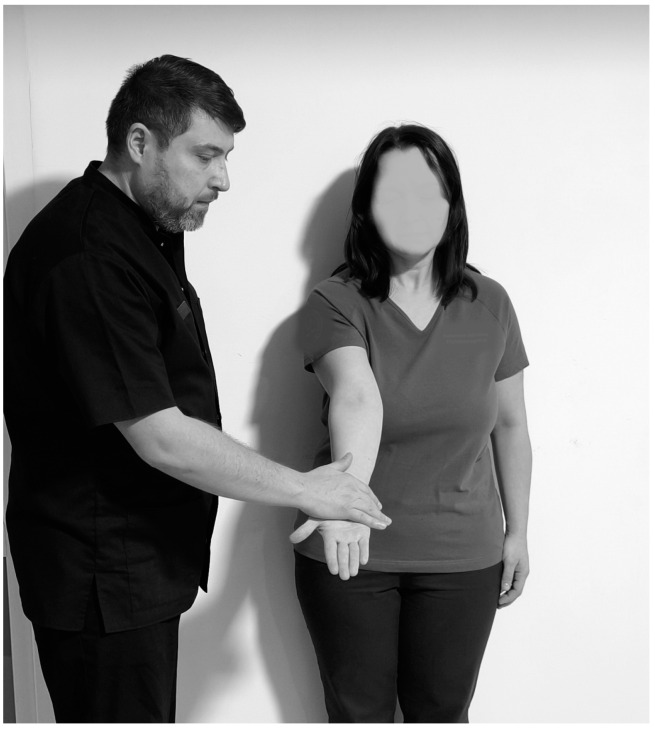
Speed test.

Another test that indicates the presence of bicipital tendonitis is the Speed test (Figure 12), which is performed with the subject in the orthostatic position. The physiotherapist stands to the side. The subject’s arm is raised to 90°, with the forearm extended from the elbow joint and the palm in supination. The physiotherapist applies a force to the distal region of the limb and the limb should maintain its position. The test is considered positive when the subject feels pain in the bicipital groove region [96,101].

### 5.12. Popeye’s Sign

Popeye’s sign is a swelling in the distal part of the arm due to biceps retraction that highlights the rupture of the long head of the bicep’s tendon [104]. The sign is discovered immediately on inspection and clinical evaluation [16].

## 6. Limitations

The limitations of this study are due to the diversity and varying diagnostic methods used in the different studies, which may lead to inconsistent data on the prevalence of specific clinical forms of SP. This inconsistency complicates the comparability of the findings. In addition, the lack of detailed demographic data, including factors such as age, gender, and activity levels, limits a comprehensive understanding of their influence on the development of SP. A significant proportion of research is based on subjective pain ratings or self-assessment, which introduces the possibility of reporting bias. In addition, diagnostic techniques—such as MRI, ultrasound, and arthroscopy—differ widely in sensitivity and specificity, which affects the accuracy of SP diagnoses. Longitudinal data on SP progression and outcomes are scarce, making it difficult to track changes over time. Finally, different definitions and classifications of types of SP across studies may reduce the consistency and reliability of epidemiologic data, further complicating efforts to draw general conclusions.

## 7. Discussion

The present study delves into the clinical forms, prevalence, and epidemiological characteristics of SP, emphasizing the complexity of their diagnosis and treatment. Each clinical form—IS, CT, Degenerative Tendinitis, BT, AC, and RCT—presents unique prevalence rates, etiologies, and clinical implications, highlighting the need for individualized management strategies.

IS, with a prevalence of 7–34%, is one of the most common SP forms. Its pathophysiology, characterized by the compression of the rotator cuff and subacromial bursa, underscores the role of mechanical factors, such as scapular instability, acromion deformity, and rotator cuff degeneration. The classification of three progressive stages—from inflammation to rotator cuff rupture—demonstrates how chronicity and delayed intervention exacerbate the condition. Notably, despite its high prevalence, 50% of individuals recover within a year with conservative management. This aligns with findings by Escamilla et al. (2014) and Dickens et al. (2005), suggesting the efficacy of physiotherapeutic interventions, especially eccentric exercises, in alleviating symptoms and restoring functionality [40,41].

CT, affecting 2.7–20% of the population, presents a unique pathology involving calcium deposition in tendons. This study reaffirms that CT predominantly affects middle-aged women, often without a history of strenuous manual labor. The progression through precalcific, calcific, and postcalcific stages emphasizes the dynamic nature of the condition, with symptom severity peaking during the resorptive phase. Diagnostic imaging, particularly ultrasound, plays a pivotal role in identifying calcifications and tracking their migration, as discussed by Gosens et al. (2009) [48]. The high prevalence of calcifications in the supraspinatus tendon further highlights the vulnerability of this anatomical structure.

The incidence of BT, closely associated with rotator cuff pathology and patient age, remains underexplored. The findings align with those of Genç and Duymaz (2020), where the Speed and Yergeson tests, complemented by imaging, confirm the diagnosis. Conservative management, including NSAIDs and physiotherapy, remains the cornerstone of treatment, emphasizing the need for early diagnosis to prevent progression [60].

AC is a debilitating condition affecting 7.1% of individuals, predominantly women aged 40–60 years. Codman’s early observations on its elusive etiology remain relevant today, as the condition often resolves spontaneously but leaves residual stiffness in a significant subset of patients. Phytotherapy and tailored physiotherapeutic interventions have shown promise in alleviating persistent symptoms, underscoring the importance of multidisciplinary approaches.

RCT exhibits an age-dependent prevalence, with 25% of individuals over 50 years affected. The findings underscore the cumulative impact of aging and mechanical stress on rotator cuff integrity. Early diagnosis through imaging, particularly MRI, remains critical, given the significant functional implications of untreated tears.

The Neer, Hawkins, Pain Arc, and Yocum tests are foundational in diagnosing IS. The Neer and Hawkins tests are particularly useful due to their high sensitivity, although the specificity can vary widely (Table 2). This variability in specificity across studies suggests that these tests alone may not conclusively diagnose IS without considering other factors or diagnostic tools, such as imaging. For instance, the Hawkins test, with moderate sensitivity and specificity, may produce false positives, especially in patients with shoulder stiffness or arthritis [95,96].

The Pain Arc test has a clear diagnostic threshold, showing that pain during shoulder abduction within a defined range suggests tendon inflammation. However, this test lacks specificity, as similar symptoms can occur in other shoulder pathologies, such as RCT or arthritis [97]. The Yocum test, with a relatively high specificity, is a reliable assessment for IS, particularly in cases where other tests may be inconclusive (Table 2).

The Jobe and Patte tests are invaluable for diagnosing RCT, particularly in the supraspinous and infraspinatus tendons. Despite their varying sensitivity and specificity, these tests are widely used due to their ease of execution and direct involvement of the affected tendon in their mechanisms (Table 2). The sensitivity and specificity of these tests are highly variable across studies, which could indicate differences in the populations tested or the way the tests are performed in clinical practice [96,98].

The Gerber test, with lower sensitivity but high specificity, is particularly useful for detecting subscapularis tendon tears (Table 2). Its utility may be limited by the subject’s physical ability to perform the test accurately, which may lead to false negatives if the subject cannot raise their hand due to muscle weakness [95,100,101].

The Coracoid Pain Test offers a novel approach to diagnosing AC by focusing on pain associated with specific anatomical areas. Its positive correlation with AC symptoms, particularly when compared to the acromioclavicular joint and subacromial regions, provides additional diagnostic confidence (Table 2). However, the reliability of this test may depend on the practitioner’s skill in palpation and the subjective nature of pain assessment [102,103].

Limited internal rotation of the glenohumeral joint serves as an early warning sign of AC (Table 2). The Glenohumeral Internal Rotation Deficit test is especially helpful in distinguishing early-stage AC from other shoulder pathologies, though it can also be influenced by other shoulder conditions like arthritis (Koester et al., 2005) [33].

Tests for BT, such as the Yagerson and Speed tests, are crucial for identifying inflammation in the bicep’s tendon. However, the low sensitivity of the Yagerson test may limit its utility in cases of early tendonitis, where symptoms might be subtle. The Speed test, while more sensitive, can also yield false positives, particularly in patients with shoulder stiffness or other underlying conditions like arthritis (Table 2) [96,101].

Popeye’s sign, an observable sign of muscle retraction due to tendon rupture, remains a valuable visual indicator for clinicians. However, it is only evident in more severe cases of tendon rupture and does not provide an early indication of tendonitis or tendinopathy [104].

## 8. Perspectives

In patients with SP (e.g., IS, RCT, AC, and BT), functional assessment using tests such as Neer, Hawkins, Pain Arc, Jobe, Patte, Gerber, Coracoid Pain Test, and Yagerson tests leads to accurate identification and diagnosis of specific shoulder disorders.

The PICO question is: In patients with shoulder pathologies (P), does functional assessment using tests such as Neer, Hawkins, Pain Arc, Jobe, Patte, Gerber, Coracoid Pain Test, and Yagerson tests (I) lead to accurate identification and diagnosis of specific shoulder disorders (O)?

Future research directions could focus on the development of standardized diagnostic criteria and tools for SP, improving consistency across studies. Clear definitions and protocols would enhance the ability to compare findings and improve diagnostic accuracy, aiding clinicians in providing better-targeted interventions for specific shoulder disorders. Additionally, conducting more extensive longitudinal studies would help track the progression of SP over time. Understanding its natural course and identifying influencing factors can provide valuable insights for predicting long-term outcomes and tailoring treatment strategies.

Combining advanced imaging techniques with machine learning could automate and improve the accuracy of SP diagnoses. Machine learning models can help identify patterns in imaging data, allowing for earlier and more accurate detection of shoulder pathologies. Gathering detailed demographic and lifestyle information, such as age, physical activity, and occupation, may help uncover risk factors linked to SP development. These data could contribute to personalized prevention strategies, enabling more effective interventions for different population groups.

Expanding research to include more diverse populations will enhance the generalizability of findings. Research conducted across a range of demographics will help create tailored prevention and intervention strategies that meet the needs of various patient profiles, improving the overall effectiveness of treatment. Incorporating these future research directions into clinical practice will lead to more personalized and effective management of shoulder pathologies, ultimately improving patient outcomes.

## 9. Conclusions

The first hypothesis was confirmed, revealing that we have identified the clinical forms of SP, with IS being the most common lesion, affecting up to 34% of individuals globally. This condition predominantly occurs in women aged 40 to 60 years.

Functional evaluation is crucial, with tests such as Neer, Hawkins, the painful arc test, and Yocum proving to be the most effective in diagnosing IS. These assessments assist specialists in distinguishing between the various clinical forms of SP and play a significant role in implementing appropriate treatment, thus confirming the second hypothesis of the research.

Functional tests are vital for evaluating and managing shoulder injuries, enabling the creation of personalized treatment plans and establishing recovery benchmarks. Tests like Neer, Hawkins, and Jobe vary in sensitivity and specificity, so clinicians must select the appropriate ones based on the patient’s condition. Enhancing patient engagement during testing is essential, as clear communication can alleviate anxiety and foster cooperation.

This study demonstrated that clinical examination combined with functional assessment remains an essential element in functional diagnosis, complementing imaging investigations that can sometimes provide false-positive or false-negative results.

Interpreting test results demands clinical judgment, particularly when different tests yield conflicting outcomes. Functional assessments should be tailored to accommodate special populations, considering any physical limitations or comorbidities. Advances in testing methods, including new technologies, continue to improve assessment accuracy and outcomes.

By integrating functional tests with other assessment techniques, such as imaging and manual muscle testing, practitioners can gain a comprehensive understanding of the patient’s condition. Ongoing professional development is essential for physiotherapists to remain current with best practices and enhance their skills. Educating patients about the purpose and significance of functional testing encourages collaboration and active involvement in their recovery process [105,106,107].

This review stands out for its multidisciplinary approach, integrating perspectives from physiotherapy and orthopedics to provide a comprehensive analysis of SP pathology, emphasizing the interplay between advanced rehabilitation techniques and surgical interventions in improving patient outcomes.

## Figures and Tables

**Table 1:** Clinical form and prevalence SP.

Clinical Form	Prevalence	Source
Impingement Syndrome	7% to 34%	Castaldo et al., 2023 [20]; Grassi et al., 2012 [21]
Calcifying Tendinitis	2.7% to 20%; Bilateral in 10% to 20% of subjects	Dudani et al., 2023 [22]; Sansone et al., 2018 [23]; Oliva et al., 2012 [24]
Degenerative Tendinitis	Incidence proportional to rotator cuff disease and patient age	Ahrens and Boileau, 2007 [25]
Bicipital tendonitis	Occurs in 5% of cases	Ahrens and Boileau, 2007 [25]
Adhesive Capsulitis	7.1%	Stella et al., 2022 [26]
Rotator Cuff Tear	25% in people over 50 years; 20% in subjects over 20 years	Minagawa et al., 2006 [27]; Yamamoto et al., 2010 [28]

**Table 2:** Clinical features and physical tests to assess patients with scapulohumeral periarthritis.

Scapulohumeral Periarthritis (SP)	Clinical Features	Physical Tests
Impingement Syndrome (IS)	- Pain during overhead activities - Limited range of motion (especially in abduction) - Tenderness over the acromion, greater tuberosity	- Neer Test: pain with arm abduction and anteversion (positive if pain occurs before full flexion) - Hawkins Test: pain with arm flexed at 90° and internal rotation - Pain Arc Test: pain between 60° and 120° of shoulder abduction - Yocum Test: pain when raising the elbow of the affected shoulder to touch the opposite shoulder
Rotator Cuff Tears (RCT)	- Weakness in shoulder movements - Pain at night - Difficulty lifting the arm overhead or performing external rotation	- Jobe Test (Empty Can Test): pain or weakness with arm abduction in the scapular plane - Patte Test: inability to hold arm in external rotation against gravity - Gerber Test (Lift-off Test): inability to lift the hand off the back suggests subscapularis tear
Adhesive Capsulitis (AC) (Frozen Shoulder)	- Severe stiffness and pain, especially in external rotation and abduction - Limited passive range of motion - Pain at night	- Coracoid Pain Test: pain upon palpation of the coracoid process, acromioclavicular joint, and anterolateral subacromial area - Glenohumeral Internal Rotation Deficit: limited passive internal rotation
Bicipital Tendonitis (BT)	- Pain in the bicipital groove, especially with overhead motions - Swelling in the upper arm - Pain on palpation of the biceps tendon	- Yagerson Test: pain with resistance during supination - Speed Test: pain in the bicipital groove when the arm is raised to 90° with palm supinated - Popeye’s Sign: visible muscle retraction indicative of rupture of the long head of the biceps tendon

## Data Availability

No new data were created or analyzed in this study.

## Supplementary Materials

The following supporting information can be downloaded at: https://www.mdpi.com/article/10.3390/life15020186/s1.

## Abbreviations

SP	Scapulohumeral periarthritis
AC	Adhesive capsulitis
OSS	Oxford Shoulder Score
QoL	Quality of life
H	Hypothesis
PRISMA	Preferred Reporting Items for Systematic Reviews and Meta-Analyses
IS	Impingement Syndrome
CT	Calcifying Tendinitis
BT	Bicipital Tendinitis
RCT	Rotator cuff tears
MRI	Magnetic resonance imaging
ROM	Range of motion
VAS	Visual Analog Scale
PICO	Patient/Population Intervention Comparison Outcomes
