# Supplementary material for: Understanding Scapulohumeral Periarthritis: A Comprehensive Systematic Review
Source: Life (Basel). 2025 Jan 26;15(2):186. doi: 10.3390/life15020186 (PMC11856014; doi:10.3390/life15020186)
Supplement: Supplementary file 1 [file life-15-00186-s001.zip › life-3395441-supplementary/life-3395441-Supplementary Materials File S1.pdf]

Search Strategies

Database 1: Medline
Search terms:

- "impingement syndrome" (MeSH Terms) OR ["impingement" (All Fields) AND "syndrome" (All Fields)]
- "calcifying tendinitis" (MeSH Terms) OR ["calcifying" (All Fields) AND "tendinitis" (All Fields)]
- "bicipital tendonitis" (MeSH Terms) OR ["bicipital" (All Fields) AND "tendonitis" (All Fields)]
- "shoulder bursitis" (MeSH Terms) OR ["shoulder" (All Fields) AND "bursitis" (All Fields)]
- "adhesive capsulitis" (MeSH Terms) OR ["adhesive" (All Fields) AND "capsulitis" (All Fields)]
- "frozen shoulder" (MeSH Terms) OR ["frozen" (All Fields) AND "shoulder" (All Fields)]
- "rotator cuff tears" (MeSH Terms) OR ["rotator" (All Fields) AND "cuff" (All Fields) AND "tears" (All Fields)]
- "scapulohumeral periarthritis" (MeSH Terms) OR ["scapulohumeral" (All Fields) AND "periarthritis" (All Fields)]
- "functional assessment" (MeSH Terms) OR ["functional" (All Fields) AND "assessment" (All Fields)]
- "clinical test" (MeSH Terms) OR ["clinical" (All Fields) AND "test" (All Fields)]

Filters applied:

- Publication date: 1972–2024
- Language: English, Russian, Swedish, German
- Article type: Cross-sectional studies, non-randomized controlled trials, randomized controlled trials, observational studies, review articles

Database 2: Pedro
Search terms:

- "impingement syndrome" OR "calcifying tendinitis" OR "bicipital tendonitis" OR "shoulder bursitis" OR "adhesive capsulitis" OR "frozen shoulder" OR "rotator cuff tears" OR "scapulohumeral periarthritis" OR "functional assessment" OR "clinical test"

Filters applied:

- Publication year: 1972–2024
- Language: English, Russian, Swedish, German

Database 3: EBSCO
Search terms:

- "impingement syndrome" AND "calcifying tendinitis" AND "bicipital tendonitis" AND "shoulder bursitis" AND "adhesive capsulitis" AND "frozen shoulder" AND "rotator cuff tears" AND "scapulohumeral periarthritis" AND "functional assessment" AND "clinical test"

Filters applied:

- Full-text availability
- Peer-reviewed articles
- Date range: 1972–2024
