# Supplementary material for: Understanding Scapulohumeral Periarthritis: A Comprehensive Systematic Review
Source: Life (Basel). 2025 Jan 26;15(2):186. doi: 10.3390/life15020186 (PMC11856014; doi:10.3390/life15020186)
Supplement: Supplementary file 1 [file life-15-00186-s001.zip › life-3395441-supplementary/life-3395441-Supplementary Materials File S2.pdf]

Theoretical background	Non-RCT (Observational, retrospective, and qualitative)	RCT study	Systematic review, meta-analysis, and review	Cross-sectional study
Rangan et al. 2015; Isaikin et al. 2027; Robinson et al. 2012; Yang et al. 2008; Mangold 2023; Grassi et al. 2012; Oliva et al. 2012; Ahrens and Boileau 2007; Habermeyer et al. 2010; Michener et al. 2003; Escamilla et al. 2014; DE Carli et al 2014; Burkhead 2011; Ricci et al. 2022; Gosens and Hofstee 2009; Gaskill and Millett 2013; Faruqi and Rizvi 2024; Conduah et al. 2010; Kuhne et al. 2009; Krupp et al. 2009; Nho et al 2010; Neviaser and Neviaser 2011; Le et al. 2017; Chambler and Carr 2003; Johnson and Robinson 2010; Kelley et al. 2013; Itoi et al. 2013; Kim et al. 2007; Nair and Kumar 2024; Jobe and Jobe 1983; Panayiotou Charalambous 2019; Szabo et al. 2023; Badau et al. 2018	Marks et al. 2019; Ekelund 1998; Abudula et al. 2024; Lyne et al. 2022; Bouaicha et al. 2020; Ganestam et al. 2015; Naredo et al. 2002; McFarland et al. 2006; Dudani et al. 2023; Minagawa et al. 2013; Yamamoto et al 2010; Karaca, 2016; Koester et al. 2005; Lin et al. 2011; Garving et al. 2017; Katthagen et al. 2016; Jonsson et. al 2006; Bernhardsson et al. 2011; Camargo et al. 2012; Gunes et al. 2017; Maier et al 2007; Hand et al. 2007; Wu et al. 2021; Stella et al. 2022; Koike et al. 2011; Stengaard et al. 2021; Neer 1972; Abate et al. 2010; Wildemann and Klatte 2012; Clement et al. 2010; Chen et al. 2003; Galatz et al 2006;Ogilvie-Harris and Wiley 1986; Dias et al 2005; Silva et al. 2008; Collin et al. 2015; Tauro 2006; Carbone et al. 2019; Mertens et al 2022;	Rangan et al. 2020; Alfredo et al. 2021; Thornton et al. 2013; Dickens et al. 2005; Liu et al. 2024; Genç and Duymaz 2020;	Moen et al. 2010; Shahabpour et al. 2008; Castaldo et al. 2023; Sansone et al. 2018; Steuri et al. 2017; Ager et al. 2020; Hirji et al 2011; Warth et al 2015; Luime et al. 2004; Malliaras et al. 2013; Neviaser and Hannafin 2010; Ryan et al. 2016; Tighe and Oakley 2008; Papadonikolakis et al. 2011; de la Serna et al. 2021; Toda 2018; Uppal et al. 2015; Hodgetts et al. 2022; Itoi et al. 2005; Via et al. 2013; Hegedus et al. 2012; Fernandez-Cuadros et al. 2019; Geaney and Mazzocca 2010;	Alqahtani et al. 2022; Alghamdi et al. 2024; Laslett et al. 2007; Stella et al. 2022; Han et al. 2021;
